# Editorial: Evidencing the impact of human-animal interaction for those living with mental health problems

**DOI:** 10.3389/fpsyt.2025.1593660

**Published:** 2025-04-10

**Authors:** Emily Shoesmith, Roxanne D. Hawkins, Elena Ratschen

**Affiliations:** ^1^ Department of Health Sciences, University of York, York, United Kingdom; ^2^ Department of Clinical and Health Psychology, University of Edinburgh, Edinburgh, United Kingdom

**Keywords:** human-animal interaction, mental health, wellbeing, animal-assisted services, animal-assisted interventions, companion animals, animals

## 1 Global mental health impact

Mental health conditions are projected to become the leading global contributors to morbidity and mortality by 2030 (1), with depression and anxiety being the most prevalent conditions (2). In the UK, the importance of identifying unmet needs and reducing health inequalities among people with mental health conditions feature prominently in National Health Service (NHS) plans (3) and strategies (4, 5) and efforts to improve services and outcomes for people with mental health conditions have been highlighted (6–8). Therefore, it is crucial to identify early preventative strategies, along with key risk and protective factors, social determinants, and the ongoing development and evaluation of therapeutic interventions in diverse contexts.

## 2 Human-animal interaction: impact, challenges and progress

The potential protective and therapeutic benefits of human-animal relationships and interactions for individuals experiencing mental health challenges (9–16) have gained increasing attention. Human-animal interaction (HAI) describes a wide spectrum of relationships and exchanges between humans and animals in a variety of contexts (17), such as in the home (e.g., companion animals, also known as untrained ‘pet’ animals), assistance animals, in therapeutic settings such as involvement in animal-assisted services (18, 19) or interaction with non-domestic animal species, whether wild or captive. However, the field of HAI frequently reports mixed results (20) and robust empirical evidence remains scarce, with existing studies often limited by methodological flaws (21–25). Key issues include small sample sizes and, consequently, lack of statistical power, lack of manualised intervention protocols, and well-designed control conditions (21, 26, 27). The majority of HAI research is correlational or small-scale, with a lack of high-quality intervention research designs capable of ascertaining causal relationships (28). Beyond observing outcomes, there is also a need for research to investigate the psychological mechanisms underlying the observed benefits and challenges associated with human-animal relationships and mental health interventions (29). While advancements have been made in enhancing methodological rigour of HAI research in recent years, substantial theoretical and practical challenges persist, hindering further progress in the field (30). Failure to advance the evidence base can lead to inefficient use of limited resources and result in poor, potentially unethical, and harmful practice for all parties involved (29).

Our Research Topic “Evidencing the Impact of Human-Animal Interaction for Those Living with Mental Health Problems”, delves into complex HAI and relationships, aiming to provide more robust empirical evidence and deepen our understanding of how HAI (which include companion animal ownership, service dogs, and animal-assisted services) can influence mental health and well-being.

Collectively, the 11 articles in this Research Topic advance our understanding of the multifaceted nature of human-animal relationships while also offering insights into the potential mental health benefits that these interactions may offer to populations with different mental health conditions. For example, several studies explored the role of animal-assisted services involving a range of animals, such as dogs, horses, and sheep. These studies reported on the role of animal-assisted services in reducing cortisol levels (Schuck et al.) and enhancing social behaviour (Nieforth et al.) in children diagnosed with Attention Deficit/Hyperactivity Disorder (ADHD); improving social functioning and self-regulation in autistic children (Peters et al.); fostering positive emotions, mindfulness, and self-efficacy in adults with substance use disorders (Schmid et al.), and alleviating symptoms of post-traumatic stress disorder (PTSD) in veterans (Rankins et al.). Placing these findings in a broader context, it is evident that HAI holds promise as a (complementary or adjunctive) intervention to improve health-related outcomes for those with mental health and/or neurodevelopmental conditions across a range of age groups.

In addition to research on animal-assisted interventions, this Research Topic also provides valuable insights into the impact of service dogs and companion animal ownership. For example, Rodriguez et al. reported that service dogs improved sleep behaviours in autistic children, and Hawkins et al. found that young adults reported positive impacts of their pet dogs and cats on their anxiety and depression symptoms, with the animals providing temporary relief during moments of interaction. Importantly, however, Hawkins et al. emphasised the need for caution, highlighting that companion animals may not always yield positive outcomes. While living with companion animals is often portrayed in the media as inherently beneficial for (mental) health (31), the complexities and potential challenges of these relationships are frequently overlooked. An additional important consideration is the potential for strong attachment to companion animals to serve as an indicator of mental health vulnerability. For example, Wells et al. suggested that a strong attachment to companion animals may correlate with personality traits typically associated with certain mental health conditions. This finding is consistent with previous literature, which has reported a negative relationship between strong emotional attachment to companion animals and mental health (32–36). While the positive effects of service dogs and companion animal ownership are evident in certain contexts, it is essential to acknowledge the potential risks and complexities associated with strong emotional attachments, which warrant further investigation.

Overall, the showcased studies emphasise the intricate and nuanced nature of human-animal relationships. While HAI may offer protective and therapeutic benefits in certain contexts, for example through hypothesised mechanisms involving attachment to or companionship provided by the animal (11, 12, 37), it is imperative to approach HAI research with a balanced perspective. Human-animal relationships may also present risks, particularly for certain populations with mental health conditions (38). For example, in addition to the findings reported in our Research Topic, previous studies have highlighted several potential challenges, which include the financial burden of animal ownership (39), the responsibility of ensuring that an animal’s needs are met (40), the grief associated with the loss of an animal (11), and the potential distress associated with the termination of animal-assisted service sessions, particularly when participants have formed an attachment to the animal (41). These factors can have significant implications for mental health.

## 3 Final considerations

The Research Topic “Evidencing the Impact of Human-Animal Interaction for Those Living with Mental Health Problems” offers a comprehensive examination of the potential benefits and complexities of HAI in mental health contexts. By presenting different research methodologies and perspectives, it underscores the importance of evidence-based approaches to integrating HAI into mental health contexts. As the field continues to evolve, future research should aim to address existing gaps, explore the long-term effects of HAI, and develop standardised protocols to maximise benefits while mitigating potential risks (20, 42, 43). In summary, while HAI present promising avenues for enhancing mental well-being, a rigorous, nuanced and evidence-based approach to research and practice will be essential to fully harness their therapeutic potential.
